# Transcriptional Profiling of *Leishmania infantum* Infected Dendritic Cells: Insights into the Role of Immunometabolism in Host-Parasite Interaction

**DOI:** 10.3390/microorganisms10071271

**Published:** 2022-06-22

**Authors:** Maritsa Margaroni, Maria Agallou, Athina Vasilakaki, Dimitra Karagkouni, Giorgos Skoufos, Artemis G. Hatzigeorgiou, Evdokia Karagouni

**Affiliations:** 1 Immunology of Infection Laboratory, Department of Microbiology, Hellenic Pasteur Institute, 11521 Athens, Greece; mmargaroni@pasteur.gr (M.M.); mariaagallou@pasteur.gr (M.A.); athens.bd@gmail.com (A.V.); 2 DIANA-Lab, Department of Computer Science and Biomedical Informatics, University of Thessaly, 35131 Lamia, Greece; dkaragkouni@uth.gr (D.K.); gskoufos@uth.gr (G.S.); arhatzig@uth.gr (A.G.H.); 3 Hellenic Pasteur Institute, 11521 Athens, Greece; 4 Department of Electrical & Computer Engineering, University of Thessaly, 38221 Volos, Greece

**Keywords:** *Leishmania infantum*, dendritic cells, transcriptome, metabolism, immune responses

## Abstract

*Leishmania* parasites are capable of effectively invading dendritic cells (DCs), a cell population orchestrating immune responses against several diseases, including leishmaniasis, by bridging innate and adaptive immunity. *Leishmania* on the other hand has evolved various mechanisms to subvert DCs activation and establish infection. Thus, the transcriptional profile of DCs derived from bone marrow (BMDCs) that have been infected with *Leishmania infantum* parasite or of DCs exposed to chemically inactivated parasites was investigated via RNA sequencing, aiming to better understand the host–pathogen interplay. Flow cytometry analysis revealed that *L. infantum* actively inhibits maturation of not only infected but also bystander BMDCs. Analysis of double-sorted *L. infantum* infected BMDCs revealed significantly increased expression of genes mainly associated with metabolism and particularly glycolysis. Moreover, differentially expressed genes (DEGs) related to DC-T cell interactions were also found to be upregulated exclusively in infected BMDCs. On the contrary, transcriptome analysis of fixed parasites containing BMDCs indicated that energy production was mediated through TCA cycle and oxidative phosphorylation. In addition, DEGs related to differentiation of DCs leading to activation and differentiation of Th17 subpopulations were detected. These findings suggest an important role of metabolism on DCs-*Leishmania* interplay and eventually disease establishment.

## 1. Introduction

Leishmaniasis is a vector-borne disease caused by intracellular parasites of the genus *Leishmania* (*L*.) and displays a variety of clinical manifestations from self-healing cutaneous to potentially fatal visceral form [1]. Visceral leishmaniasis is endemic mainly in tropical, subtropical and Mediterranean countries. According to WHO, 310 million people are at risk, about 90,000 are diagnosed with visceral leishmaniasis per year, while the number of deaths is estimated to reach 30,000 annually [2]. *Leishmania* parasites are dimorphic organisms. In the sandfly, the flagellated motile form called promastigote progresses through several morphological stages of differentiation and finally it becomes the non-dividing metacyclic promastigote, the infective form of the parasite that is transmitted from the sandfly to the mammalian host. These parasites are eventually phagocytosed by host’s phagocytes as macrophages [3] and dendritic cells (DCs) [4].

DCs are professional antigen-presenting cells that orchestrate the initiation of immunological responses and the induction of tolerance [5]. In the case of infection, they sense pathogens in their environment through pattern recognition receptors and transport antigens from infected tissue to draining secondary lymphatic organs, where they prime naïve T cells thus linking innate and adaptive immunity [6,7]. Importantly, distinct differentiation stages of DCs are involved in the development of different types of effector T cells or the induction of regulatory T lymphocytes that in turn inhibit the activation of effector T cells. The ability of DCs to drive polarization of different T helper (Th) subsets has been suggested to be orchestrated by the engagement of distinct metabolic pathways [8]. Recent literature shows that infection of DCs with different pathogens results in increasing energy demands, both anabolic and catabolic, driving the cells to use alternative metabolic pathways to support their immune regulatory role [9]. Specifically, differentiated DCs that are capable of priming and activating T cells are dependent on a switch from oxidative phosphorylation to glycolysis [10,11,12,13,14,15,16,17,18,19], mediated by several molecules such as the transcription factor 1a induced by hypoxia (HIF-1a) whose role is to promote the transcription of different enzymes involved with glucose metabolism [20]. 

The interaction between *Leishmania* and DCs via the involvement of different molecules and the participation of multiple signaling pathways leads up to phenotypic and functional alterations in DCs. These changes affect the proper cytokine production by DCs and eventually the activation of T cells which control parasite infection [4]. Thus, it has been shown that different *Leishmania* species affect the immune system in different ways, i.e., inhibition of DCs’ activation, to facilitate their survival inside the host. One strategy is the efficient parasite uptake by DC allowing the pathogen to hide from immune recognition and subsequently to downregulate immune responses by subverting DC signaling, gene expression and immune activation in the effort to successfully establish infection (reviewed in [21] and [22]). Previously, we and others have shown that DCs harboring *L. infantum* parasites are characterized by an immature phenotype and reduced activation, migration properties as well as antigen-presentation capacity. Importantly, these DCs are able to produce the anti-inflammatory cytokine IL-10 driving the differentiation and activation of regulatory T cells that are responsible for visceral disease establishment [23,24]. However, due to the complexity of the interplay between DCs and the parasite, the way the parasite manipulates DCs function to favor survival and replication is not clear yet. 

Systems biology, through high throughput technologies, may contribute to the elucidation of host pathogen interactions. Microarray analyses have examined global changes in gene expression of macrophages and dendritic cells in response to different *Leishmania* species [25,26,27,28]. RNA sequencing, which allows complete coverage of transcriptome, could be an effective tool in investigating the interplay between *Leishmania* and host cells. Until recently, the majority of transcriptome studies concerned macrophages infected with dermotoropic species, i.e., *L. amazonensis*, *L. major* and *L. panamensis* [29,30]. Τhere are few studies concerning dendritic cells and *L. amazonensis* [31,32], whereas no information to our knowledge is available regarding dendritic cells infected with *L. infantum.*

To extend the knowledge regarding the molecular events involved in the systemic immune response to *L. infantum* infection, we conducted transcriptomics using bone marrow derived DCs (BMDCs) that have been likened to in vivo murine iDCs [33]. Comparison of uninfected BMDCs versus infected ones resulted in a very significant increase in glycolytic enzymes along with surface molecules expression that drive T cell exhaustion. On the contrary, BMDCs harboring fixed *L. infantum* promastigotes showed predisposition to TCA cycle followed by a mature phenotype. 

## 2. Materials and Methods

### 2.1. Ethics Statement

Animal usage complied to PD 86/2020-A’ 199 and European Directive 2010/63/EU, and was based on 3 + 1R: Replacement, Reduction, Refinement and Respect. The experimental protocol has been approved by the Institutional Protocol Evaluation Committee and the Official Veterinary Authorities of Attiki’s Prefecture under the license 6381/11-12-2017. Animals’ welfare was assessed by licensed users. Proper actions were adopted to minimize animal pain. 

### 2.2. Mice

For the purposes of the study female BALB/c mice were used at the age of 6–8 weeks old and maintained under SPF (Specific Pathogens Free) conditions at the authorized animal facilities in Hellenic Pasteur Institute (HPI). Animals were housed under standard environmental conditions for this species and were provided with a diet of appropriate food pellets and water ad libitum.

### 2.3. Parasites

*L. infantum* (MHOM/GR/2001/GH8) strain [34], was cultured in vitro at 26 °C in complete RPMI-1640 (Biowest). The medium was supplemented with L-glutamine (2 mM), HEPES (10 mM), NaHCO3 (24 mM), penicillin (100 U/mL), streptomycin (10 µg/mL) and 10% (*v*/*v*) heat-inactivated fetal bovine serum (FBS; Biowest, Riverside, MO, USA). All experiments were conducted using promastigotes till passage five. *L. infantum* parasites maintained infectivity through serial passage in BALB/c mice. Wherever applicable, stationary phase promastigotes were CFSE-labeled (Invitrogen, Carlsbad, CA, USA) according to a protocol applied by Resende et al. [24]. In some cases, stationary phase promastigotes, CFSE-labeled or not, were fixed by exposing them to 0.1% glutarhaldehyde for 10 min. 

### 2.4. Generation of Bone Marrow-Derived Dendritic Cells

DCs were differentiated from murine bone marrow of BALB/c mice according to a protocol described by Lutz et al. [35] and will be referred as BMDCs. On day 8, non- and semi-adherent cells were harvested and phenotypically assayed by staining with PE-conjugated anti-mouse CD11c monoclonal antibody (Table 1). Routinely, the purity of BMDCs cells was superior to 75% and eventually they were used for all the assays described.

### 2.5. In Vitro BMDCs Infection with L. infantum Parasites

On day 8 of culture, BMDCs were collected and seeded in a 24-well plate at a density of 1 × 10^6^/mL. For sorting experiments, BMDCs were seeded in 100 mm Petri dishes. Cells were exposed to live (vGH8) or glutaraldehyde-fixed stationary phase *L. infantum* promastigotes (fGH8) (wherever applicable parasites were previously stained with CFSE) at a ratio of infection 20:1 followed by gentle pipetting to facilitate contact among BMDCs and parasites. Four hours later, non-phagocytosed parasites were removed by gently washing, fresh complete culture medium was added to the wells, and cells were cultured for a total of 24 h. Evaluation of BMDCs infection was conducted with flow cytometry through determination of CFSE^+^ BMDCs. Otherwise, cells were Giemsa stained and the infection of BMDCs was determined microscopically.

### 2.6. BMDCs Phenotypic Analysis by Flow Cytometry

BMDCs placed in 24-well plates (1 × 10^6^ cells/mL) were infected with CFSE-labeled *L. infantum* stationary phase promastigotes or glutaraldehyde-fixed parasites, as described above. Untreated or BMDCs treated with LPS (1 µg/mL) served as negative and positive controls, respectively. Twenty-four hours later, BMDCs were washed with FACS buffer (PBS—3% (*v*/*v*) FBS), labelled with R-PE-conjugated anti-mouse CD40, CD80, CD86, CD200 or CD273 mAbs (Table 1) and incubated in the dark for 30 min at 4 °C. Following staining and washing, cells were run on a FACS Calibur system (Becton-Dickinson, San Jose, CA, USA) equipped with CellQuest software. BMDCs were detected based on phenotypic data (FSC-SSC parameters) and on expression levels of CD11c molecule. CaliBRITE™ beads (BD Biosciences, Erembodegem, Belgium) were used for instrument calibration. All data acquired were processed with FlowJo software version 10.0 (Tree Star Inc., Ashland, OR, USA).

### 2.7. BMDCs-Induced T Cell Proliferation Assay

BMDCs seeded in a 96-well U-bottom plate (4 × 10^5^ cells/mL) were cultured in the presence of *L. infantum* stationary phase promastigotes at a ratio of DCs:parasite 1:20 as described in paragraph 2.5. After washing non-internalized parasites, previously isolated CD4^+^ or CD8^+^ T cells from spleens of naïve BALB/c mice were put in contact with BMDCs at a ratio 5:1 for 96 h. At the final 18 h of culture, cells were pulsed with 0.5 µCi of [^3^H]-thymidine. CD4^+^ or CD8^+^ T cells isolation was conducted with Dynabeads™ Untouched™ Mouse CD4 or CD8 Cells Kit (ThermoFischer Scientific, Rockford, IL, USA) according to instructions of the manufacturer. Then, cells were harvested and the proliferation of CD4^+^ or CD8^+^ T cells was measured by [^3^H]-TdR incorporation with a microplate scintillation counter (Microbeta Trilux, Wallac, Turcu, Finland). All samples were run in triplicate and results are expressed as stimulation index (SI: S.I. = cpm measured in T cells co-cultured with *L. infantum*-infected BMDCs/cpm in T cells co-cultured with naïve BMDCs).

### 2.8. Sorting of Infected or Fixed Parasite-Exposed BMDCs

For RNA extraction experiments, sorting of infected or fixed parasite-exposed BMDCs was required. For this purpose, BMDCs were exposed to CFSE-labelled live or glutaraldehyde-fixed *L. infantum* stationary phase promastigotes. Twenty-four hours later, CD11c^+^ in the case of non-infected or CFSE^+^CD11c^+^ BMDCS in the case of infected or fixed parasite-exposed cells were sorted. Briefly, cells were washed with FACS buffer (PBS—3% (*v*/*v*) FBS) twice and were stained at a density of 50 × 10^6^ cells/mL with PE-conjugated anti-CD11c monoclonal antibody (dilution 1:100). Then, BMDCs were resuspended in PBS containing 1 mM EDTA and 1% FBS at a density of 20 × 10^6^ cells/mL. Cell strainers (40 mm) (Falcon) were used for dissociation of cell aggregates. Subsequently, cells were placed on ice until the sorting was conducted. Sorting of the BMDCs was performed with the help of FACSAria (BD Biosciences, San Jose, CA, USA). Single cells were selected on SSC-H/SSC-W, and FSC-H/FSC-W dot plots. *L. infantum*-infected BMDCs were chosen for sorting by selecting cells double positive for surface CD11c and CFSE fluorescence with the BD FACSDiva^TM^ software (BD Biosciences) in polypropylene tubes (BD Biosciences) previously coated with FBS (18 h at 4 °C). Routinely, the sorting efficiency was >95%. FACSAria performance was assessed using Cytometer Setup and Tracking Beads (CS&T beads, BD Biosciences, Erembodegem, Belgium) and Accudrop technology was implemented to set drop delay value during cell sorting. 

### 2.9. Isolation of RNA, Construction of Library and Sequencing

The isolation of RNA was conducted in two independent biological replicates of non-infected BMDCs and three independent biological replicates of infected or fixed parasite-exposed BMDCs with the use of the Trizol Reagent (Invitrogen, Carlsbad, CA, USA). Qiagen RNeasy mini kit (Qiagen, Hilden, Germany) was used for RNA purification. Concentration and quality control of the isolated RNA were assessed using the ND-1000 Nanodrop microspectophotometer (ThermoFisher Scientific, Wilmington, DE, USA) and the 6000 Nano LabChip kit on the Agilent Bioanalyzer 2100 (Agilent Technologies, Inc., Palo Alto, CA, USA). Only RNA samples with RIN number > 7.5 were used for RNA Sequencing (RNA-Seq) using Ion Torrent technology.

### 2.10. RNA Sequencing Data Analysis and Further Downstream Analysis

Transcript level quantification of RNA-Seq samples was performed using Salmon version 1.2.1 [36]. Mouse transcriptome was compiled from GENCODE [37]. Differential Expression Analysis (DEA) was conducted using EdgeR [38], employing quasi-likelihood F-tests. Gene set enrichment analysis (GSEA) of differentially expressed genes (DEGs) was conducted for KEGG pathways [39] and Gene Ontology term downstream analysis [40,41] using the R packages limma [42] and clusterProfiler [43,44], respectively. Gene set enrichment analysis was performed separately for up- and downregulated genes. Benjamini-Hochberg false discovery rate was used for the correction of enrichment *p* values for multiple comparisons and a 0.01 *p*-value threshold was applied. REVIGO was further applied to simplify the list of GO enriched terms obtained by discarding terms with a dispensability value above 0.7 [45]. Visualization of targeted pathway members in KEGG pathways was obtained with Pathview. Network analysis was derived from the String Database [46] using 7 types of evidence. In particular, experiments, text mining, gene fusion, neighborhood, co-expression, databases and co-occurrence as active interaction sources were selected for conducting network analysis. Figure 3, Figures S1, S4 and S5 were produced using R versions 3.6 and 4.0 [47] and ggplot2 [48].

### 2.11. Statistical Analysis

The results were expressed as mean ± standard deviation (SD). GraphPad Prism version 6.0 software (San Diego, CA, USA) was used for conducting statistical analysis. One-way ANOVA with multiple comparisons Tukey–Kramer post hoc test was applied and the value of *p* < 0.05 was considered to be significant for all analyses. 

## 3. Results

### 3.1. Leishmania infantum Parasites Actively Inhibit BMDCs Maturation

**Figure 1:**
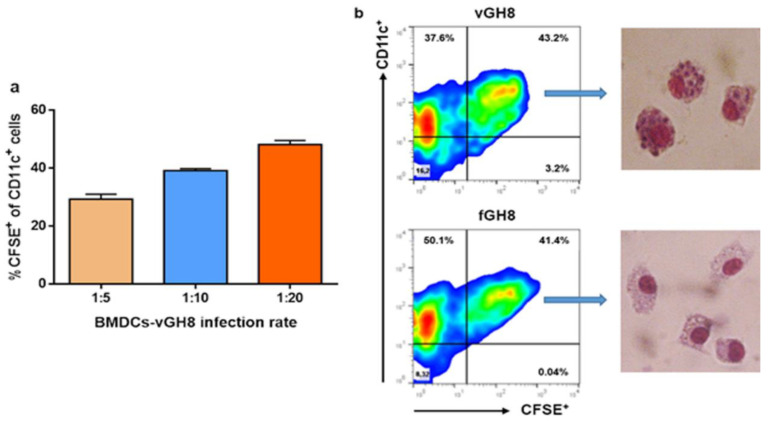
Evaluation of BMDCs’ ability to internalize the promastigote form of *L. infantum* parasites: (**a**) *L. infantum* promastigotes stained with CFSE were added to BMDCs in vitro cultures at different infection ratios, i.e., 1:5, 1:10, 1:20 and sampled at 24 h in order to assess the best ratio by flow cytometry analyses. Percentages of CFSE^+^ BMDCs were estimated. The mean ± SD of three independent experiments is shown. (**b**) Representative flow cytometry analyses plots at infection ratio of 1:20 for *L. infantum*-infected and fixed parasite-harboring BMDCs are shown with the respective Giemsa-stained microscopic analyses in the selected populations.

Due to controversial results in previous studies regarding DCs infection by *Leishmania* parasites [23,49,50,51,52], we first examined the ability of BMDCs to internalize the promastigote forms of *L*. *infantum* parasites in our experimental system. For this purpose, BMDCs were put in contact with CFSE-labeled parasites at different DCs:parasites ratio, i.e., 1:5, 1:10 and 1:20 for 24 h. CFSE-labeled glutaraldehyde-fixed parasites were used as a control for assessing the potential of BMDCs to internalize dead parasites and for investigating whether parasite internalization is an active process. Flow cytometry analyses documented that BMDCs could effectively phagocytose promastigote forms of the parasite with the highest infection rate detected at 1:20 ratio where about 50% of BMDCs had been infected (Figure 1a). The success of infection was determined further by the effective transformation of *L*. *infantum* promastigotes into amastigotes, the intracellular form of the parasite, inside host BMDCs obtained from parallel in vitro cultures stained with Giemsa (Figure 1b). Interestingly, a similar number of BMDCs were found to be CFSE^+^ after exposure to glutaraldehyde-fixed parasites, supporting the premise that they were also able to phagocytose fixed parasites (Figure 1b). However, microscopic evaluation revealed that BMDCs did not contain any parasite form after a 24 h exposure to fixed parasites (Figure 1b). Thus, the detected fluorescence may have been the result of parasite degradation inside host cell. 

**Figure 2:**
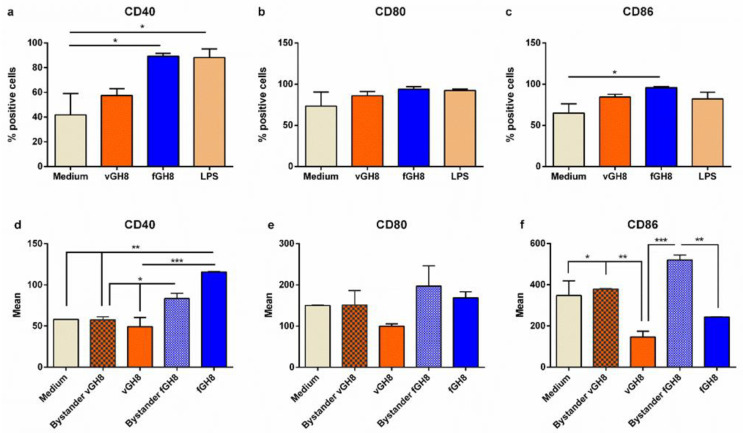
Evaluation of maturation of *L*. *infantum*-infected BMDCs and fixed parasite-harboring BMDCs. Populations expressing: (**a**) CD40, (**b**) CD80 and (**c**) CD86 co-stimulatory molecules were detected by flow cytometry. (**d**–**f**) The surface co-stimulatory molecules were further analyzed in bystander and *L*. *infantum*-infected BMDCs and fixed parasite-harboring BMDCs expressed as Mean (mean fluorescence). The mean ± SD of three independent experiments is shown (*: *p* < 0.05, ** *p* < 0.01, ***: *p* < 0.001).

Subsequently, we investigated the effect of *L*. *infantum* amastigotes settlement into BMDCs, through assessment of BMDCs maturation. For this purpose, the expression of CD40, CD80 and CD86 co-stimulatory molecules was determined by flow cytometry with a special focus on BMDCs harboring *L. infantum* amastigotes or having phagocytozed fixed parasites. For each molecule examined, we first analyzed the entire BMDCs populations, irrespective of CFSE staining. Moreover, for the purpose of experimentation, LPS was used as a positive control of BMDCs maturation. Unlike that of LPS, the uptake of *Leishmania* parasites by BMDCs did not obviously upregulate co-stimulatory molecules expression (Figure 2a–c). On the contrary, phagocytosis of fixed *L. infantum* promastigotes induced significant upregulation of CD40 and CD86 molecules, and a slight non-significant increase in CD80 molecule (Figure 2a–c). To characterize the uninfected (bystander) and the infected BMDCs found in the population, results were further analyzed based on CFSE^+^ gating. It must be noted that the percentage of *L*. *infantum*-infected (CFSE^+^) BMDCs expressing CD80 or CD86 were lower compared to naïve BMDCs whereas CD40 expression levels in this population were the same, thus confirming parasites’ stealthy entry (Figure 2d–f). Importantly, the bystander BMDCs population (CFSE^−^) of infected BMDCs did not also exhibit a mature phenotype suggesting that the microenvironment produced by the infected BMDCs negatively affected non-infected bystander BMDCs activation (Figure 2d–f). Similar analyses were performed on BMDCs populations incubated with fixed *Leishmania* parasites. After 24 h of incubation, fixed parasite-harboring BMDCs, as well as their bystander populations, expressed high levels of CD40 molecules (Figure 2d,e). However, in the case of CD80 and CD86, only the bystander population expressed elevated levels of these molecules suggesting that the fixed parasites uptake did not actively induce its expression, whereas the microenvironment created by those cells induced CD80 and CD86 expression in the bystander population (Figure 2f). 

Overall, *Leishmania* may directly interact with BMDCs to induce weak maturation in a minority of cells also affecting the bystander populations by inhibiting their maturation. On the other hand, the interactions between BMDCs and fixed *Leishmania* parasites resulted in strong expression in both bystander and fixed parasites-harboring populations.

### 3.2. Transcriptome-Wide Profiles of Leishmania-Infected and Fixed Parasites-Harboring BMDCs

**Figure 3:**
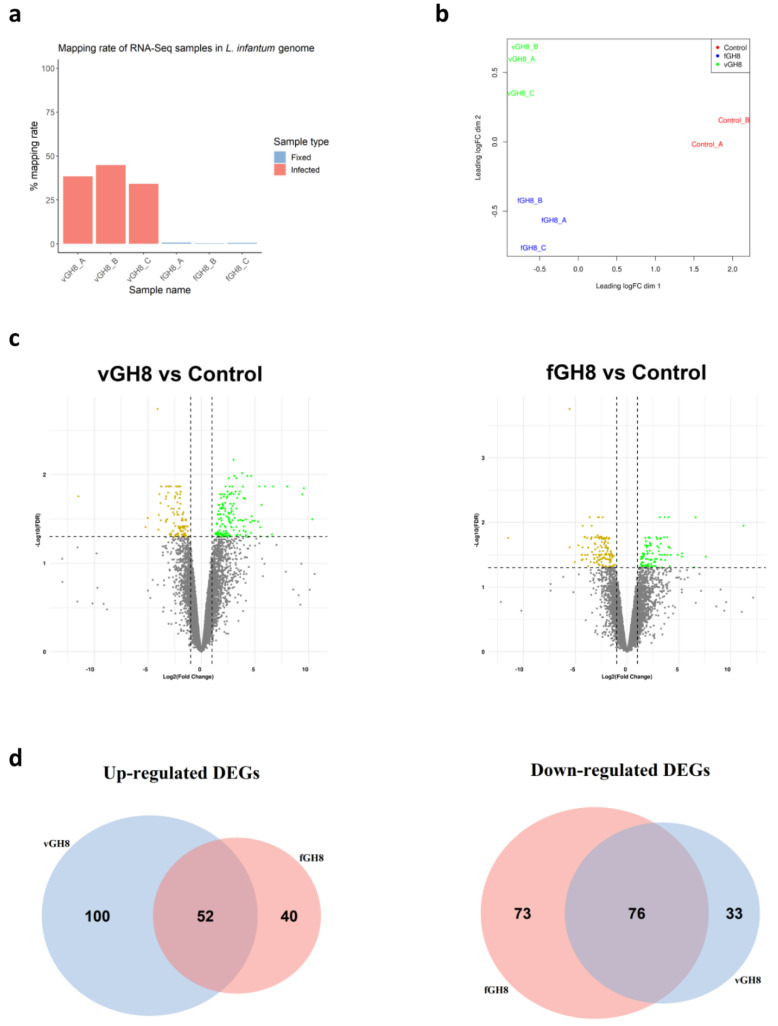
Identification of differentially expressed genes (DEGs) in *Leishmania*-infected BMDCs (vGH8) and BMDCs harboring fixed parasites (fGH8): (**a**) Mapping rates of RNA-seq samples in *L. infantum* genome; (**b**) Multidimensional scaling (MDS) plot of DEGs in *Leishmania*-infected BMDCs (vGH8), BMDCs harboring fixed parasites (fGH8) and naïve BMDCs (Control); (**c**) Volcano plots showing differential expression analysis between vGH8 versus Control and fGH8 versus Control BMDCs. Up- and downregulated genes (FDR < 0.05) are depicted with green and yellow, respectively; (**d**) Venn diagrams portraying the number of exclusively and common up- (left) and downregulated (right) genes for each comparison.

In order to examine the transcriptional changes that accompanied BMDCs maturation during parasite uptake, special attention was given to specifically sort only BMDCs that harbored parasites (virulent or fixed). The purpose of this was to avoid taking into account uninfected bystander DC populations along with *Leishmania*-hosting BMDCs. Flow cytometry analysis of BMDCs with gating on forward and side scatter and CD11c expression demonstrated that both live and fixed parasites-harboring BMDCs could be detected and distinguished by the presence of CFSE-labelled amastigotes. Thus, with fluorescence-activated cell sorting, we were able to isolate infected BMDCs or BMDCs harboring fixed parasites despite their relative low numbers. Subsequently, total RNA from these populations was extracted for library construction and sequencing. The RNA-seq generated ~25 million paired-end 95-bp sequencing reads per sample. Within the sequencing libraries, reads were about equally distributed among the host genome (average: 61%, range: 55.2 to 65.8%) and the *L. infantum* genome (average: 39%, range: 38.4 to 44.8%) (Figure 3a). As expected, in the fixed-parasites containing BMDCs, reads were mapped to the mouse reference genome (average: 99.5%, range: 99.3 to 99.7%), whereas only an average of 0.5% of reads mapped to the *L. infantum* genome indicating that fixed parasites were effectively processed (Figure 3a). After exclusion of reads mapped against the *L. infantum* genome, quantification of mouse transcriptome was conducted with Salmon version 1.2.1. Multidimensional scaling (MDS) plot showed that *Leishmania*-exposed (virulent or fixed) and non-infected BMDCs were found at the left and right corner of the plot, respectively, implying different gene regulation to naïve BMDCs (Figure 3b). However, the separation between *Leishmania*-infected and fixed parasite-containing BMDCs was less prominent with the respective populations to cluster towards the lower and upper ends of the second dimension (Figure 3b). Unsupervised hierarchical clustering of expression data also verified the segregation of samples by state (Figure S1). Linear model-based statistical analysis identified an equal number of DEGs (FDR < 0.05), 261 and 241 for *Leishmania*-infected BMDCs and BMDCs containing fixed parasites, respectively, compared to naïve BMDCs (Figure 3c). Out of these DEGs, 152 (~58%) were upregulated by virulent parasite in contrast to 92 DE (~38%) found to be upregulated by fixed parasite. Conversely, 109 (~42%) and 149 DE (~62%) were downregulated by virulent and fixed-parasites, respectively. Of all these DEGs, 133 were exclusively modulated by the virulent parasite, 100 DE genes (~75%) were upregulated and only 33 DE genes (~25%) were downregulated. However, of the 113 exclusive DE genes modulated by the fixed parasite, only 40 genes were upregulated and 76 were downregulated. One hundred and twenty-eight were commonly regulated by both parasites with 52 (~41%) genes upregulated and 76 (~59%) genes downregulated (Figure 3d). 

### 3.3. L. infantum Infection Affects Genes Regulating the Metabolism and Immune Response of BMDCs

**Figure 4:**
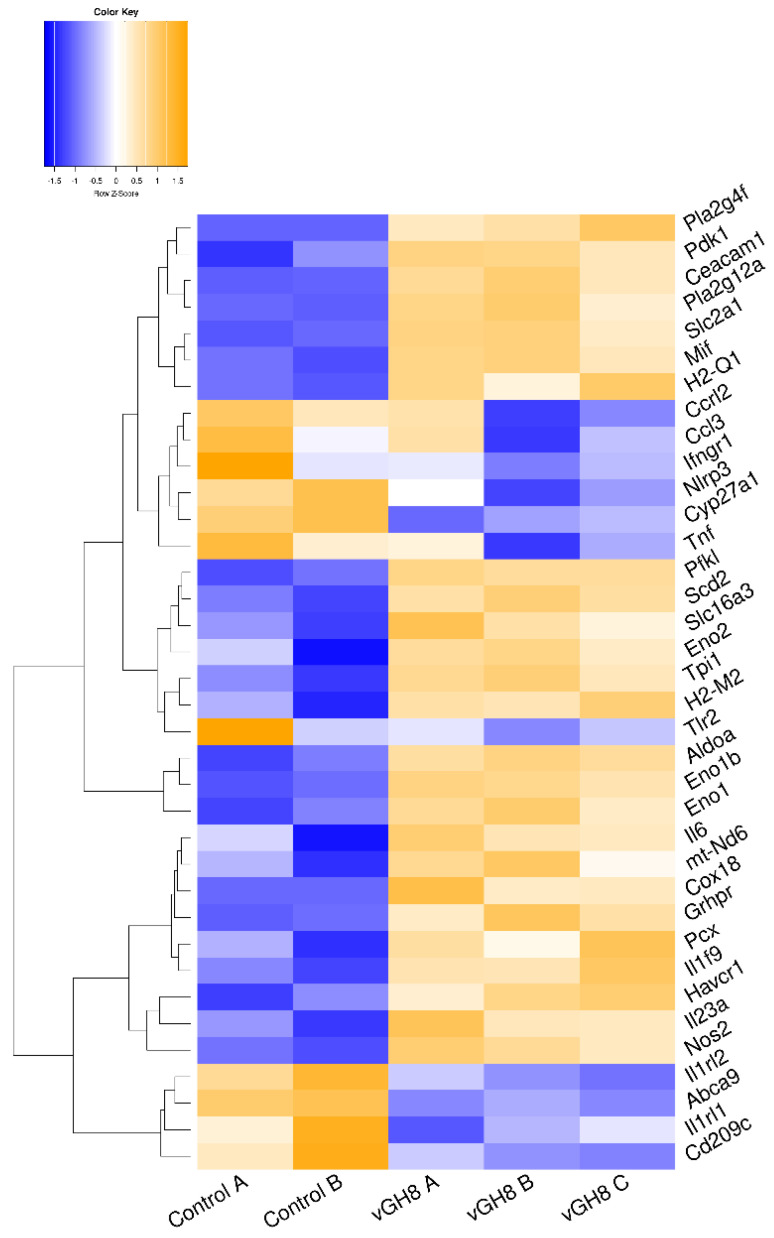
Unsupervised hierarchical clustering based on expression of metabolism-related DEGs between vGH8 versus Control BMDCs.

STRING analysis for the identified DEGs in infected BMDCs showed a high degree of connectivity between them. Specifically, an independent k-means clustering analysis identified an enrichment of host cell genes encoding molecules involved in metabolic pathways and immune response (Figure S2). Regarding metabolism, the majority of those genes encoded enzymes participating in the glycolysis pathway. Specifically, infection induced expression of genes encoding glucose transporter GLUT1 (slc2a1) and monocarboxylate transporter MCT4 (slc16a3). Furthermore, several glycolytic enzymes such as phosphofructokinase (pfkl), fructose-biphosphate aldolase a (aldoa), enolase 1, enolase 1b and enolase 2 (eno1, eno1b, eno2) and triose phosphate isomerase 1 (tpi1) were induced (Figure 4, File S1A). Importantly, we detected that *L. infantum* infection induced upregulation of the transcript encoding glyoxylate/hydroxypyruvate reductase (Grhpr), an enzyme promoting the metabolism of pyruvate to lactate, as well as the transcript Pdk1 that encodes for the pyruvate dehydrogenase kinase isoenzyme 1, which inactivates pyruvate dehydrogenase (Figure 4, File S1A). It is known that pyruvate dehydrogenase metabolizes pyruvate—the end product of glycolysis—to acetyl-CoA. However, in the pyruvate dehydrogenase’s absence, pyruvate is metabolized into lactate via activation of lactate dehydrogenase, further suggesting that in this case *L. infantum* infection stimulated DCs metabolism shift to aerobic glycolysis. Downstream in this pathway, we observed increased levels of the gene that encodes for stearoyl-CoA desaturase 2 (scd2). This enzyme is responsible for converting stearoyl-CoA to oleyl-CoA and thus regulating monounsaturated: saturated fatty acids ratio. Moreover, we detected downregulation of “ATP-binding cassette transporter A9” encoding gene (abca9) that holds a key role in oxysterol production and thus in inhibition of cholesterol accumulation via mediating the efflux of the sterol 27-hydroxylase (cyp27a1) along with cellular cholesterol and phospholipids (Figure 4, File S1A). Thus, the parasite may modulate the biosynthesis of fatty acids while it enhances cholesterol uptake accompanied by decreased cholesterol efflux, increasing the possibility of cholesterol accumulation within infected BMDCs. Further, we detected a significant increase in the genes encoding the Cytosolic Phospholipase A2 Zeta (Pla2g4f) as well as secreted Phospholipase A2 (Pla2g12a) suggesting that *Leishmania* parasite induced the biosynthesis of the arachidonic acid (AA) cascade involved in the release of polyunsaturated fatty acids (Figure 4, File S1A). 

It has been shown that in order for the BMDCs to be activated, they have to incorporate pyruvate into their mitochondrial TCA cycle. Otherwise, TCA prevention limits BMDCs maturation and cytokine production upon microbial stimulation resulting in induction of immune responses mediated by regulatory or Th17 rather than Th1 and Th2 populations (reviewed in [8]). In agreement with this, in *L. infantum*-infected BMDCs, only a few genes encoding for immunology-related genes were detected. These were involved in DCs-T cells interaction through antigen presentation via MHC class I and II molecules, as evidenced by the increased expression of H2-Q1 and H2-M molecules (H2-M2), respectively, as well as induction of T cell tolerance with increased levels of genes encoding for HAVCR1 and CEACAM1 surface molecules. 

Moreover, there was significant downregulation of the gene encoding DC-SIGN (cd209c). DC-SIGN is a lectin that holds an important role in DCs-neutrophil crosstalk and migration of DCs through binding to ICAM-2 which is located in the blood and lymphatic vascular bed (Figure 4, File S1A). The downmodulation of DC-SIGN may further result to abortive interactions among DCs and T cells, since the DC-SIGN binding to lymphocyte ICAM-3 could be hampered. Consistent with this was also the reduced transcription level of ifngr1 that encodes the receptor for IFNγ. Surprisingly, modulation of cytokine genes was restricted to increased levels of molecules involved in dendritic cells activation leading to a pro-inflammatory response. Specifically, we detected increased levels of il1f9, mif and inos genes encoding IL-36γ cytokine, MIF chemokine and iNOS, respectively (Figure S2 and Figure 4, File S1A). 

Importantly, Venn diagrams revealed that the aforementioned molecules were exclusively up- or downregulated, in *Leishmania*-infected BMDCs. Taken together, the above data suggest that the immune-related genes regulation could prevent T cells from proper activation, thus impeding their proliferation and their development to end-stage lymphocytes demonstrating either effector or regulatory functions.

**Figure 5:**
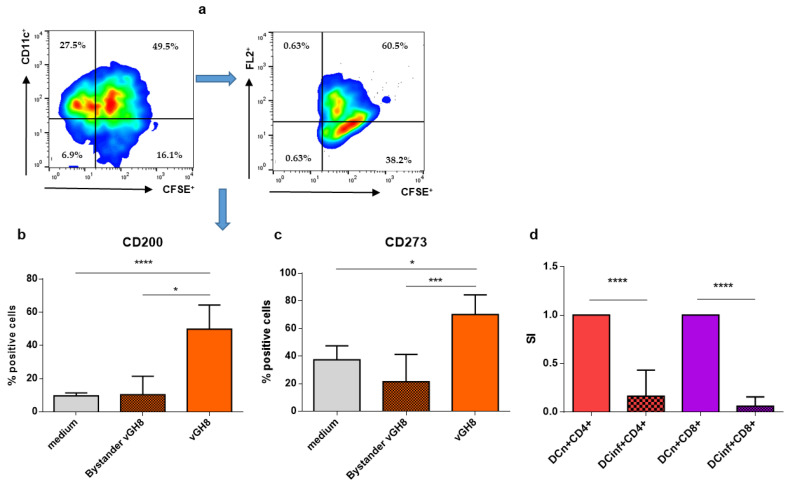
Effect of *L. infantum* infection of BMDCs on the expression of immune checkpoint molecules and on T cell proliferation/activation: (**a**) Representative dot plots of the gating strategy to isolate/discriminate CD200 and CD273 (PD-L2) expressing parasite-infected BMDCs from by-stander BMDCs. (**b**) CD200 and (**c**) CD273 molecules were detected in bystander and *L. infantum*-infected BMDCs by flow cytometry. (**d**) BMDCs exposed to *L. infantum* were co-cultured with CD4^+^ or CD8^+^ T cells isolated from spleen obtained from a naïve mouse for 96 h. The final 18 h, cultures were pulsed with 0.5 µCi of [^3^H]-TdR. The mean ± SD of three independent experiments is shown (*: *p* < 0.05, ***: *p* < 0.001, ****: *p* < 0.0001).

In order to further characterize *Leishmania*-infected BMDCs, the expression of CD200 and CD273, also known as immune checkpoint molecules, was assessed. Flow cytometry analysis revealed that infection of BMDCs with vGH8 resulted in statistically increased expression of CD200 and CD273 on BMDCs’ surface (Figure 5a). The signaling pathways associated with these immune checkpoint molecules are associated with the phenomenon of T cell exhaustion. The expression of CD200 (Figure 5b) and CD273 (Figure 5c) was found exclusively in infected and not in bystander BMDCs, indicating that infected cells could not affect neighboring bystander BMDCs. The impaired DC-T cell interaction was also supported by the finding that CD4^+^ and CD8^+^ T cells co-cultured with *L. infantum*-infected BMDCs showed significantly decreased proliferation capacity compared to those co-cultured with BMDCs that had not been exposed to the parasite, indicating T cell exhaustion (Figure 5d). 

### 3.4. Fixed Parasites Induced the Activation of BMDCs into Th17-Promoting Cells

**Figure 6:**
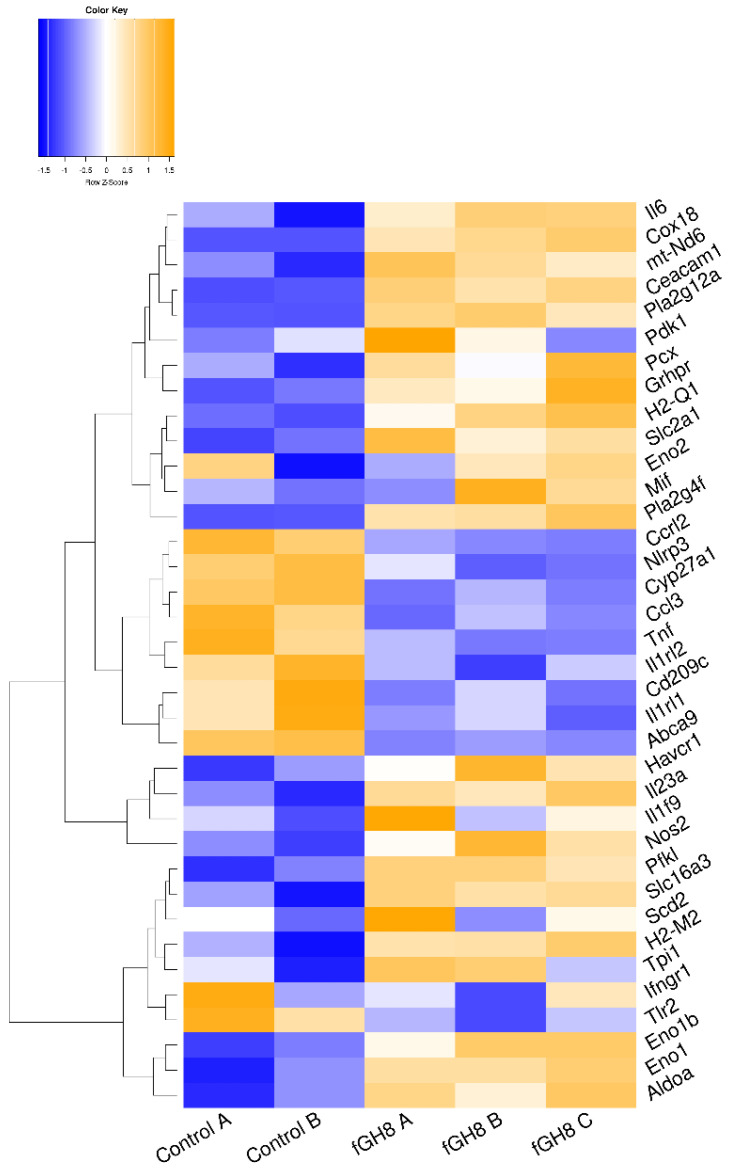
Unsupervised hierarchical clustering based on expression of metabolism-related DEGs between fGH8 versus Control BMDCs.

Similar to STRING analysis of *Leishmania*-infected BMDCs, analysis of fixed parasites-harboring BMDCs revealed the existence of two clusters with a high degree of connectivity between them. These clusters contained DEGs that were involved mainly in immune response (Figure S3). In fact, transcriptome analysis of fixed parasites containing BMDCs suggested that phagocytosis of fixed parasites by BMDCs induced energy production through TCA cycle and oxidative phosphorylation. This was proven by the upregulated transcript encoding pyruvate carboxylase (Pcx) that leads pyruvate to be metabolized to acetyl-CoA through TCA cycle (Figure 6, File S1B). Moreover, the expression of NADH:Ubiquinone oxidoreductase core Subunit 6 (mt-Nd6) as well as cytochrome c oxidase assembly protein 18 (Cox18) that belong to NADH dehydrogenase (Complex I) and complex IV of the mitochondrial membrane respiratory chain, respectively, further supported this (Figure 6, File S1B). Moreover, we detected similar downregulation of abca9 and cyp27a1 as well as increased levels of Pla2g4 as well as Pla2g12a that belong to lipid biosynthesis pathways compared to *L. infantum*-infected BMDCs (Figure 6, File S1B). Interestingly, internalization of fixed parasites clearly did not induce the expression of enzymes associated with the glycolysis pathway, suggesting that only the live parasite actively regulated the induction of anaerobic glycolysis metabolism.

Among the up-regulated DEGs, those encoding for pro-inflammatory IL-6 and IL-23 cytokines, which act synergistically for Th17 cells differentiation were listed, supporting the activation of BMDCs (Figure 6, File S1B). Importantly, genes that are actively involved in inflammatory reactions and thus BMDCs activation such as Ccl3, Tnf, Ifngr1, Nlrp3, Ccrl2, Tlr2, Il1rl1 and Il1rl2, were significantly downregulated (Figure 6, File S1B). Thus, these data potentially suggest the terminal differentiation of BMDCs to cell populations driving differentiation of T cells to Th17 populations.

### 3.5. GO Analysis

**Figure 7:**
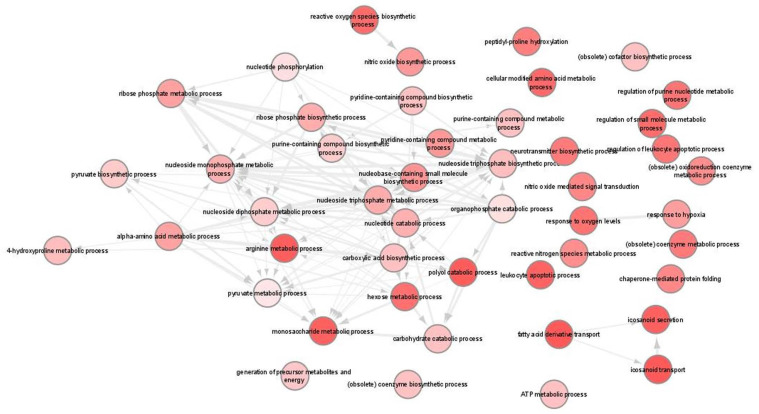
Gene ontology (GO) enrichment analysis in *L. infantum* infected BMDCs summarized by REVIGO. Graph shows biological processes enriched (parents GO terms) in upregulated genes (Benjamini-Hochberg *p*-value < 0.01). Highly similar GO terms are linked by edges. Bubble color indicates the user-provided *p*-value. GO terms that are highly similar are linked by edges in the graph, where the degree of similarity is indicated by the line width [45].

We next used Gene Ontology (GO) systems of classification to decipher how the cellular responses evoked against infection. This analysis resulted in 93 enriched GO terms and applied to upregulated genes only (File S2A). REVIGO was used to further reduce these terms to a non-redundant set of 47 representative terms (Figure 7). Results revealed significant enrichment of terms associated with pyruvate, nucleotide and amino acid metabolic processes as well as the response to hypoxia and nitric oxide biosynthetic process. Moreover, KEGG pathway analysis revealed only five pathways that were related to “HIF-1 signaling pathway”, “Glycolysis/Gluconeogenesis”, “Biosynthesis of amino acids”, “Central carbon metabolism in cancer” and “Carbon metabolism” (Table 2; Figures S4 and S5).

**Figure 8:**
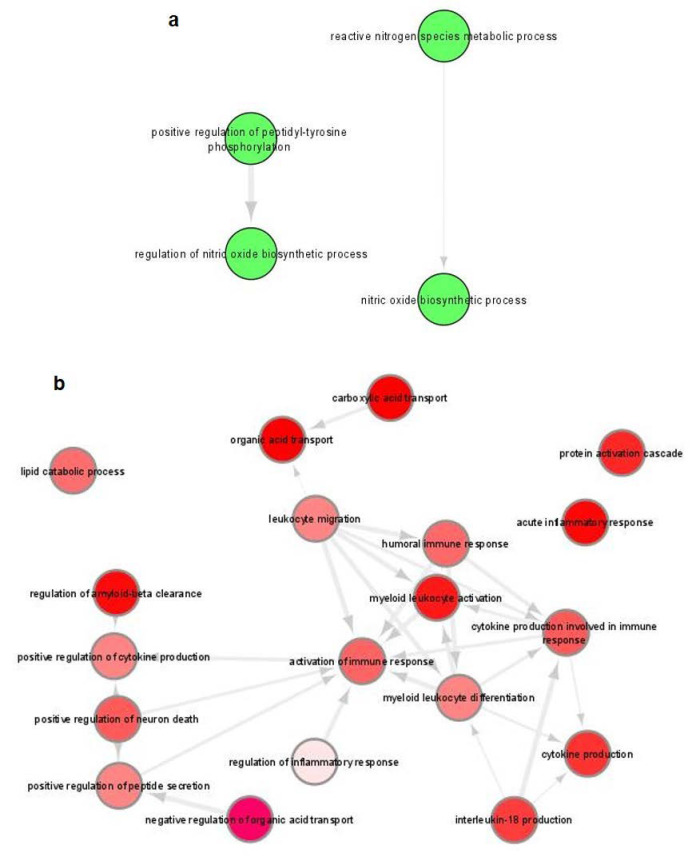
Gene ontology (GO) enrichment analysis of DEGs in fixed parasites-harboring BMDCs summarized by REVIGO. Graph shows biological processes enriched (parents GO terms) in: (**a**) upregulated and (**b**) downregulated genes (Benjamini-Hochberg *p*-value < 0.01). Highly similar GO terms are linked by edges. Bubble color indicates the user-provided *p*-value. GO terms that are highly similar are linked by edges in the graph, where the degree of similarity is indicated by the line width [45].

GO terms in fixed parasites-containing BMDCs were significantly enriched for both the up- and downregulated set of genes, while the identified terms were associated with immune responses and infectious diseases (File S2B,C). Specifically, GO terms of upregulated DEGs were associated with “Positive regulation of peptidyl-tyrosine phosphorylation” and “Nitric oxide biosynthetic process” (Figure 8a), whereas terms of downregulated DEGs were associated with functions such as regulation of inflammation, cytokine production and leukocyte migration (Figure 8b). On the contrary, KEGG pathways were significantly enriched only for the downregulated DEGs and were associated with “Chagas disease”, “Lysosome”, “Other glycan degradation”, “C-type lectin receptor signaling pathway” and “Malaria” (Table 3).

## 4. Discussion

*Leishmania* parasites and host antigen-presenting cells interactions are quite perplexing and hold a key role on the final outcome of leishmaniasis. The early response of DCs infected with *Leishmania* parasites and how this profoundly impacts on the subsequent adaptive immune response is of high interest, based on DCs role in growth and survival of the parasite and the eventual susceptibility of the host [53,54,55,56,57,58]. In the present study, the ability of BMDCs to internalize *L. infantum* was investigated by incubating BMDCs with live parasites at different ratios for 24 h. Our results showed that almost 50% of BMDCs were effectively infected by *L. infantum* promastigotes as evidenced by the successful transformation to amastigotes, the intracellular form of the parasite. These data were in accordance with previous studies concerning the infection rate of BMDCs exposed to *L. infantum* [53,55]. Furthermore, co-culture of BMDCs with promastigote forms of dermotropic species of the parasite, such as *L. major*, *L. amazonensis* and *L. braziliensis* resulted in similar rates of infectivity with parasites being able to survive and multiply inside BMDCs [53,58,59]. 

As DCs are known to orchestrate the immune response to infection, it was of high interest to study the effect of virulent compared to fixed *L. infantum* promastigotes internalization on BMDCs’ maturation. Phagocytosis of virulent parasites from BMDCs inhibited their maturation, also accompanied by inhibition of bystander population’s maturation. Similarly, it has been shown that *L. infantum* infection of BMDCs induces TNFα and IL-10 production, whereas IL-12 production and CD40 expression remained unaffected compared to non-infected cells [23]. Moreover, Neves et al. supported that *L. infantum* promastigotes do not induce upregulation of CD40 and CD86 expression [55]. However, in the case of human DCs, *L. infantum* and *L. braziliensis*, as well as *L. amazonensis* are capable of inducing an increase in CD86 expression [53]. Interestingly, fixed parasites induced strong expression of all co-stimulatory molecules tested, implying that active infection hampered DCs activation, which piqued our interest to shed light on DCs–parasite interaction and the pathways used by the parasite to overcome the host’s defense mechanisms by applying RNA-seq.

Since only 50% of the BMDCs were infected by *L. infantum* parasites in our system, this could result in diluted biological signals due to the presence of uninfected DCs. To overcome this major challenge, we isolated double–sorted (CD11c^+^CFSE^+^) BMDCs by flow cytometry and determined global gene expression pattern in total RNA isolated exclusively from double sorted populations. 

Our findings showed that among the reads generated from the whole sample, 60% of them were mapped to the mouse genome and 40% to the parasitic genome, contrary to fixed-harboring DCs, where about 90% of the reads were mapped to the mouse genome verifying the active infection. In macrophages infected with *L. donovani* parasites, 67% of reads mapped to the mouse genome and this percentage may depict the proportion of RNA molecules from mouse or parasite [60]. It is noteworthy that among the deregulated genes in the two groups, 52 genes were commonly upregulated and 76 were downregulated, while a larger number of genes was exclusively upregulated in infected BMDCs compared to those downregulated, indicating that infection activates BMDCs. 

Gene set enrichment analysis in our experimental system revealed that transcriptome of *L. infantum*-infected BMDCs, 24 h post infection, was significantly enriched in upregulated genes encoding for molecules such as glucose, slc2a1, pfkl, pdk1 and grhpr, suggesting a metabolism shift to aerobic glycolysis. Glycolysis is a basic metabolic process that in some cases produces energy in the form of ATP and NADH via conversion of glucose into pyruvate. This phenomenon is similar to the Warburg effect on cancer cells [61]. During Warburg metabolism, an increased glucose uptake is conducted by the cell, generating pyruvate and other glycolytic intermediates storage that are used to meet the need in energy wanted for cell proliferation [62]. Macrophages and BMDCs undergo metabolic changes in response to environmental stimuli [12,14,15,63,64]. The effect, though, of intracellular pathogens on cellular metabolism is characterized by high complexity and has not been studied in depth, since cells of the host undergo metabolic changes either to directly kill pathogens or to restrict their access to essential nutrients [65,66]. On the other hand, pathogens have evolved mechanisms to subvert host cell metabolism for their own benefit. It has been shown that anabolic and catabolic demands of DCs are increased when cells have to adapt in an inflammatory milieu caused by infection. In such a case, redirection of certain metabolic pathways is observed in order to support DCs functions [9]. Typically, DCs meet the need for energy supply and biomolecules synthesis through oxidative phosphorylation and oxidation of fatty acids [67]. Recent evidence suggests that DCs alter their metabolic program to anaerobic glycolysis, characterized by conversion of pyruvate to lactate, once the pathogen is recognized via PAMP molecules [11,15].

In the same context as our findings, increased transcription of aldoa, pfk and eno2 has been observed in macrophages infected with *L. major*, 3 h post infection, supporting the activation of aerobic glycolysis in these cells [28]. The difference in the timepoint of metabolism switching, compared to our results, could possibly be attributed to the different role of DCs compared to that of macrophages. Similarly, *L. infantum* induces a significant increase in pfk and pdk1 genes exclusively in infected and not in bystander bone marrow macrophages, also demonstrating a rapid swift to aerobic glycolysis [68]. Moreover, in accordance with pervious findings by Rabhi et al. and Moreira et al. the swift to aerobic glycolysis is induced only by live parasites and not by those chemically inactivated suggesting the active role of the parasite in the host’s metabolism subversion [28,68]. 

Regarding other pathogens, a study from Hardgrave et al., supports that infection of BMDCs with *Toxoplasma gondii*, an obligate intracellular parasite, induces aerobic glycolysis in host cells [69]. Bacterial infections are also known for shifting host cell’s metabolism. Similarly to our findings, *Mycobacterium tuberculosis* infection of macrophages induces upregulation of glycolytic enzymes and transporters that are essential for glucose uptake [70]. Moreover, dendritic cells infected with active influenza A virus show a metabolic switching that plays an important role in immune response to infection [71]. Furthermore, human dendritic cells stimulated with *Aspergillus fumigatus* are characterized by enhanced uptake of glucose resulting in increased lactate release, implying glycolysis induction [13]. As a conclusion, all the above data suggest that switching to aerobic glycolysis is a common mechanism observed in DCs infected with pathogens.

Recently, a key role has been attributed to HIF-1a regarding the induction of glycolysis, since it regulates the expression of several enzymes that play a role in glucose metabolism [20]. In the experimental model of chronic visceral leishmaniasis, it has been shown that HIF-1a contributes to *L. donovani* infection establishment, through abrogation of IL-12 production by splenic DCs leading to limited expansion of the Th1 cell [72]. In our experimental system, GO enrichment and KEGG analysis in BMDCs infected by *L. infantum* revealed significant enrichment of terms and pathways associated with hypoxia, HIF-1 signaling and Glycolysis/Gluconeogenesis, highlighting the role of these pathways in infection. Hammami et al. demonstrated the important role of the IRF-5/HIF 1a transcription factor axis in the prevention of CD8^+^ T cells expansion by DCs [73], whereas regarding iNOS, it has been shown that when expressed under HIF-1a axis, it inhibits OXPHOS through the produced NO, thus resulting in the impairment of GM-DCs to stimulate T cells in the long term [12].

Except from glycolysis induction, we found that *L. infantum* favored the synthesis of saturated fatty acids, while genes preventing cholesterol accumulation were downregulated raising the possibility of cholesterol accumulation. It has previously been shown that *L. major* infection of macrophages inhibits the efflux of cholesterol in the genome level and induces triacylglycerides synthesis suggesting a disturbance of cholesterol homeostasis that may lead to cholesterol accumulation and foam cell formation [28]. Among the de-regulated genes found in our study was cyp27a1 that is known to be involved in degradation of cholesterol by promoting fluid membrane formation that leads to defective antigen presentation [74,75]. The disruption of host membrane structure by the *Leishmania* parasite results in altered IFNγ receptor conformation and thus in defective IFNγ signaling with compromised antileishmanial activity [76], as evidenced in our results by downregulated Ifngr1 expression. Moreover, *Leishmania*, by decreasing cholesterol in the host cells’ membrane, alters the conformation of MHC II, possibly contributing to a faster dissociation of the peptide to be presented, hampering antigen specific T cell activation [77]. Furthermore, membrane dissociation also leads to CD40 miss-localization into the non-graft regions, enhancing *Leishmania* infection mediated by IL-10 rather than controlling IL-12 production [78]. This mechanism may be related to inhibition of BMDCs maturation after infection with *Leishmania,* also found in our study to affect T cell activation and the subsequent immune response to the parasite. 

Defective antigen-presentation of infected BMDCs to T cells resulting in inhibition of their proper activation was further evidenced by the finding that *L. infantum* affected the transcription of only a small number of immune-related genes, among which are genes associated with T cell tolerance, such as HAVCR1, also known as Tim-1, CEACAM1 and DC-SIGN. Tim-1′s signal transduction has been associated with increased IL-4 production by differentiated Th2 cells [79], while the immunosuppressive capacity of CEACAM1 is exploited by several pathogens of bacterial and viral origin, since CEACAM-specific adhesins allow these pathogens to attach, invade and colonize host cells [80]. DC-SIGN, abundantly expressed by DCs in vitro and in vivo, mediates adhesion with T cells by binding ICAM-3 with high affinity [81]. Thus, the *Leishmania*-induced downmodulation of DC-SIGN impairs DCs-T cell interaction.

Importantly, co-culture of either CD4^+^ or CD8^+^ T cells with *L. infantum*-infected BMDCs resulted in their defective activation shown by their inability to proliferate, which may be the result of the acquired tolerogenic phenotype of BMDCs, characterized by increased expression levels of CD200 and CD273 molecules. It has been shown that among the strategies that *Leishmania* parasites have evolved to undermine host defense mechanisms is the induction of CD200-CD200R signaling pathway. CD200 and CD200R molecules are involved in the downregulation of myeloid and lymphoid cells via the inhibition of mitogen kinases such as PI3K and ERK (reviewed in [82]). Cortez et al., have demonstrated that *L. amazonensis* induces the expression of CD200 molecule in host macrophages favoring parasite’s replication [83]. CD273, also known as PD-L2, is a ligand of PD-1 molecule mainly expressed in APCs. Engagement of PD-1 by PD-L2 results in inhibition of proliferation and cytokine production by CD4^+^ T cells mediated via T cell receptor (TCR) [84]. Importantly, in clinical VL, exhausted populations have been detected, mainly consisted of CD8^+^ T cells and less by CD4^+^ T cells [85]. Thus, in keeping with the literature, our results demonstrate that the increased expression of both CD200 and CD273 molecules in surface of *L. infantum*-infected BMDCs further supported the acquisition of a suppressive phenotype.

Paradoxically, along with the suppressor molecules we found increased levels of mif, il1 and il36 expression. Regarding pro-inflammatory cytokines IL-1 and IL-36, these are associated with the onset of an effective immune response against *Leishmania* infection and are represented by Th1 profile [86]. Moreover, MIF chemokine prevents activation-induced apoptosis and promotes sustained expression of inflammatory factors such as TNF-α and nitric oxide, acting as a survival factor [87], while when it is expressed under HIF-1a axis it promotes *L. amazonensis* killing under hypoxic conditions [88]. The above findings reflect the constant battle between the host and the parasite for dominion over each other, which does not always end with only one winner.

On the other hand, fixed *L. infantum* entrance in BMDCs favored OXPHOS for energy production. Moreover, DEGs in BMDCs hosting fixed parasites suggested that these cells lead to differentiation of T cells into Th17 subpopulation by the increased levels of il6 and il23 genes found. It is known that IL-6 along with TGF-β induce the development and maintenance of Th17 effector cells through upregulation of the IL-23 cytokine and its receptors [89], which have been associated with protection against visceral leishmaniasis [90].

## 5. Conclusions

In conclusion, our findings support the notion that *Leishmania* parasites affect DCs phenotype by hampering maturation and increasing the expression of immune checkpoint molecules eliciting suppressive immune responses. Moreover, genes exclusively increased in *L. infantum*-infected BMDCs imply a metabolism rewiring towards glycolysis. On the contrary, genes exclusively deregulated in BMDCs harboring glutaraldehyde-fixed *L. infantum* were mainly involved in inflammatory reactions suggesting differentiation to Th17 populations. A full elucidation of the role of immunometabolism in DCs–*Leishmania* interplay can propose candidate molecules as new therapeutic interventions against leishmaniasis. 

## Figures and Tables

**Table 1:** Antibodies used for phenotypic analysis by flow cytometry.

Antibody	Fluorochrom Conjugate	Clone	Dilution/Concentration	Company	City and Country of Production
CD11c	PE	HL3	1/100	BD Biosciences	Erembodegem, Belgium
CD40	R-PE	3/23	1/100	BD Biosciences	Erembodegem, Belgium
CD80	R-PE	16-10A1	1/100	BD Biosciences	Erembodegem, Belgium
CD86	R-PE	GL	1/100	BD Biosciences	Erembodegem, Belgium
CD200	PE	OX90	0.25 µg/test	Invitrogen	San Diego, CA, USA
CD273	PE	TY25	0.06 µg/test	Invitrogen	San Diego, CA, USA

**Table 2:** KEGG pathways enriched by upregulated DEGs in *L. infantum*-infected BM-DCs.

KEGG Pathway	Gene Symbol	Adjusted *p* Value
mmu04066~HIF-1 signaling pathway	Pfkl, Eno2, Slc2a1, Egln3, Eno1b, Nos2, Pgk1, Eno1, Egln1, Aldoa, Pdk1	4.3 × 10^−7^
mmu00010~Glycolysis/Gluconeogenesis	Pfkl, Eno2, Pgm1, Tpi1, Pgk1, Eno1, Aldoa, Pkm	4.3 × 10^−7^
mmu01230~Biosynthesis of amino acids	Pfkl, Eno2, Eno1b, Tpi1, Pgk1, Eno1, Aldoa, Ass1, Pkm	1.28 × 10^−6^
mmu05230~Central carbon metabolism in cancer	Pfkl, Slc2a1, Pdgfrb, Slc16a3, Pdk1, Fgfr3, Pkm	1.01 × 10^−4^
mmu01200~Carbon metabolism	Pfkl, Eno2, Eno1b, Tpi1, Pgk1, Eno1, Aldoa, Pkm	4.24 × 10^−4^

**Table 3:** KEGG pathways enriched by downregulated DEGs in *L*. *infantum*-infected BM-DCs.

KEGG Pathway	Gene Symbol	Adjusted *p* Value
mmu05142: Chagas disease	Ifngr1, Ccl3, Gna15, C1qa, Mapk14, Tnf, 1qc, Tlr2	0.0006
mmu04142: Lysosome	Man2b1, Ctse, Cd164, Hexb, Gm2a, Ctsf, Glb1, Fuca1	0.0014
mmu00511: Other glycan degradation	Man2b1, Hexb, Glb1, Fuca1	0.0014
mmu04625: C-type lectin receptor signaling pathway	Clec4b1, Cd209c, Mapk14, Tnf, Nlrp3, Egr2, Pak1	0.0031
mmu05144: Malaria	Klrb1a, Tnf, Klrb1b, Tlr2, Itgal	0.0069

## Data Availability

The data discussed in this publication have been deposited in NCBI’s Gene Expression Omnibus [91] and are accessible through GEO Series accession number GSE204807 (https://www.ncbi.nlm.nih.gov/geo/query/acc.cgi?acc=GSE204807 (accessed on 25 May 2022)).

## Supplementary Materials

The following supporting information can be downloaded at: https://www.mdpi.com/article/10.3390/microorganisms10071271/s1. Figure S1. Unsupervised hierarchical clustering based on expression of DEGs between vGH8 versus Control and fGH8 versus Control BMDCs. Figure S2. STRING cluster analyses of DEGs in *L*. *infantum*-infected BMDCs. Gray lines correspond to active interactions. Figure S3. STRING cluster analyses of DEGs in fixed parasite-harboring BMDCs. Gray lines correspond to active interactions. Figure S4. Glycolysis/Gluconeogenesis signaling pathway. Glycolysis/Gluconeogenesis pathway present in the KEGG database was significantly enriched in upregulated genes of *L. infantum*-infected DCs versus control cells. Benjamini–Hochberg false discovery rate was used for correction of enrichment *p* values and a threshold of *p*-value < 0.01 was applied. Visualization of KEGG pathways and targeted members was conducted with R package Pathview. Figure S5. HIF1 signaling pathway. HIF1 signaling pathway present in the KEGG database was significantly enriched in upregulated genes of *L. infantum*-infected DCs versus control cells. Benjamini–Hochberg false discovery rate was used for correction of enrichment *p* values and a *p*-value < 0.01 threshold was applied. Visualization of KEGG pathways and targeted members was conducted with R package Pathview. File S1. Differentially expressed genes (DEGs) in: (A) *Leishmania*-infected BMDCs and (B) in BMDCs harboring fixed parasites. File S2. Gene Ontology (GO) enrichment analysis of DCs exposed to *L. infantum* parasites. (A) Significantly enriched GO terms in upregulated genes in infected DCs. Significantly enriched GO terms in (B) upregulated and (C) downregulated genes in DCs harboring fixed parasites.
