# Supplementary figures and images for: Transcriptional Profiling of Leishmania infantum Infected Dendritic Cells: Insights into the Role of Immunometabolism in Host-Parasite Interaction
Source: Microorganisms. 2022 Jun 22;10(7):1271. doi: 10.3390/microorganisms10071271 (PMC9322131; doi:10.3390/microorganisms10071271)

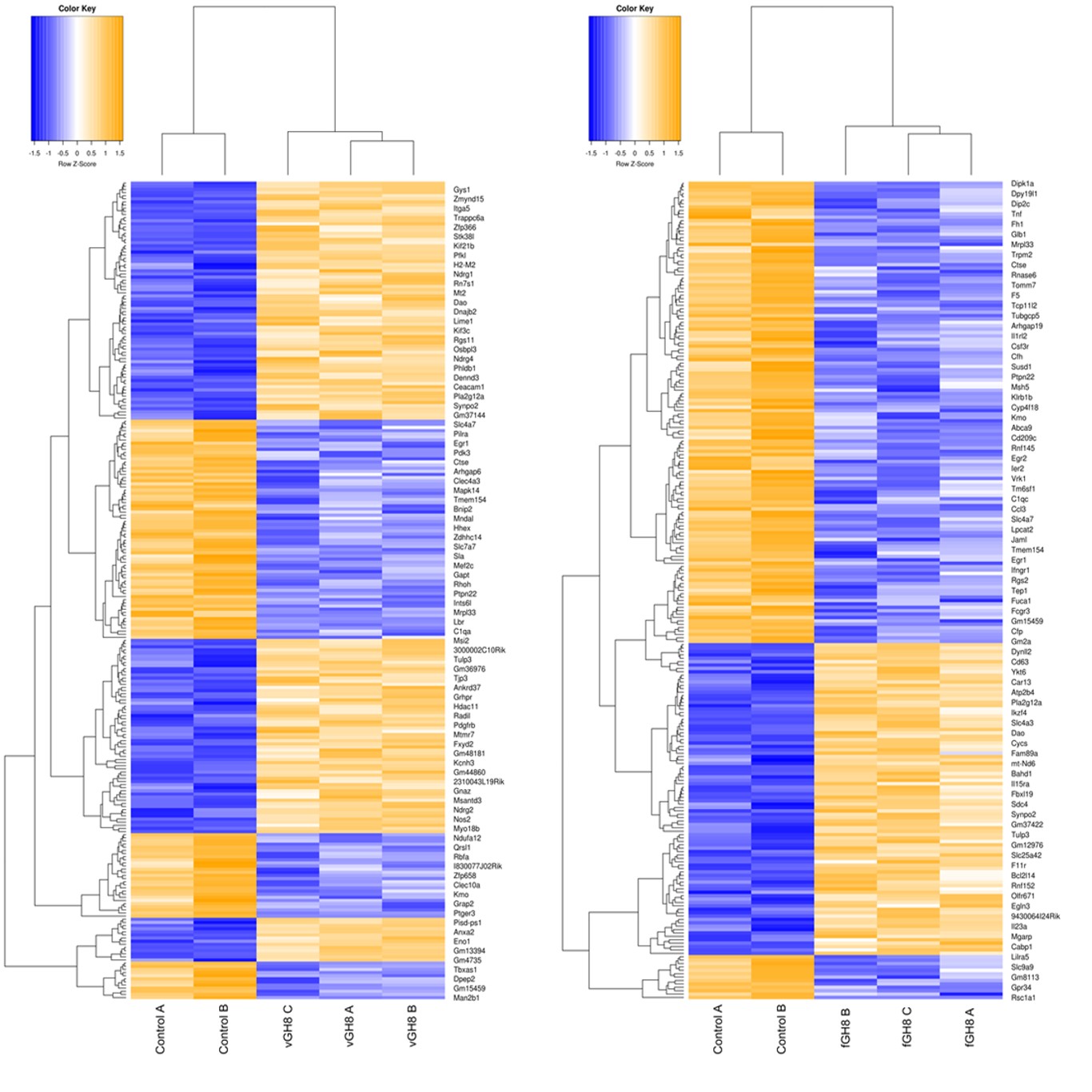

Supplement: Supplementary file 1 [file microorganisms-10-01271-s001.zip › microorganisms-1767867-supplementary/Figure S1.jpg]

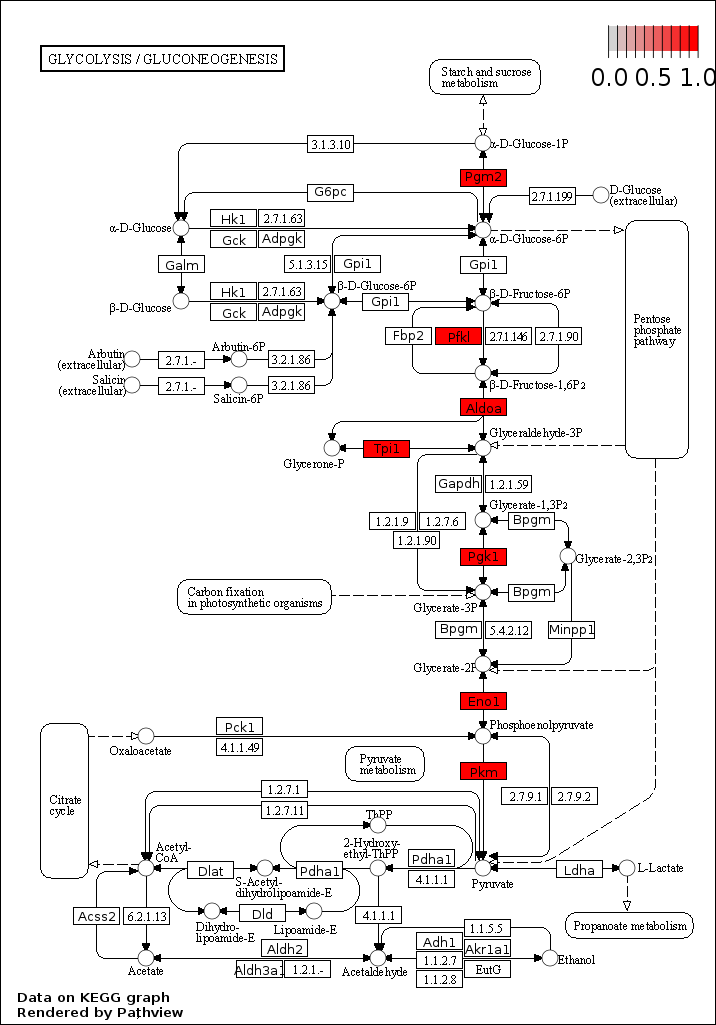

Supplement: Supplementary file 1 [file microorganisms-10-01271-s001.zip › microorganisms-1767867-supplementary/Figure S4.tif]

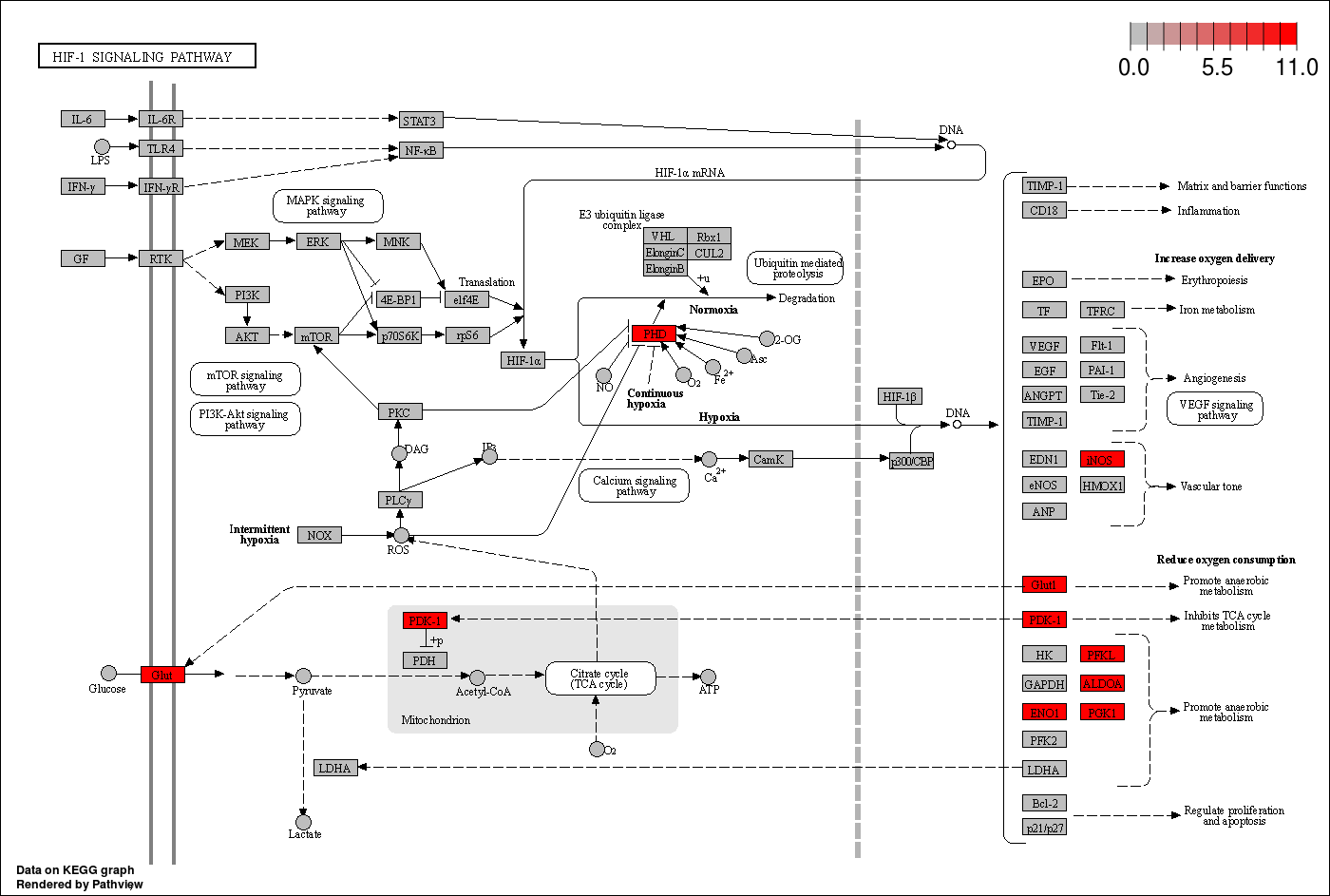

Supplement: Supplementary file 1 [file microorganisms-10-01271-s001.zip › microorganisms-1767867-supplementary/Figure S5.tif]
